# Effect of nutraceutical supplementation on semen quality in stallions

**DOI:** 10.1002/vms3.1289

**Published:** 2023-10-10

**Authors:** Edgar Díaz Rojas, Dalia I. Carrillo Moreno, Viridiana Contreras Villarreal, Fernando Arellano Rodríguez, Alan S. Alvarado Espino, Oscar Ángel García

**Affiliations:** ^1^ Ciencias en Producción Agropecuaria Universidad Autónoma Agraria Antonio Narro Torreón Coahuila Mexico; ^2^ Departamento de Producción Animal Universidad Autónoma Agraria Antonio Narro Torreón Coahuila Mexico; ^3^ Departamento de Ciencias Médico Veterinarias Universidad Autónoma Agraria Antonio Narro Torreón Coahuila Mexico

**Keywords:** horses, nutraceutical, nutrition, semen quality, supplementation

## Abstract

**Background:**

The use of reproductive biotechnologies in equine practice has shown that some stallions are subfertile, so ways to improve fertility have been sought.

**Objective:**

This study aimed to evaluate the effect of nutraceutical supplementation on improving semen quality in Quarter Horse stallions.

**Methods:**

Semen from six Quarter Horse stallions was assessed for 4 months every 20 days using the computer‐assisted semen analysis system. They were evaluated for 60 days before supplementation; then, the same stallions were re‐evaluated for 60 days with nutraceutical supplementation (30 g/day).

**Results:**

Volume showed no significant difference (*p* > 0.05) with nutraceuticals. Sperm concentration (10x^6^) was significantly higher with supplementation (339.4 ± 17.5 sperm/mL) than without supplementation (224.6 ± 19.9). Sperm abnormalities (%) were significantly (*p* < 0.05) lower with supplementation (14.3 ± 0.6) than without supplementation (19.1 ± 0.4). Sperm kinematic parameters, total motility (TM), progressive motility (PM), rectilinear velocity (VSL), the velocity of the trajectory (VAP) and curvilinear velocity (VCL), were significantly better with supplementation (*p* < 0.05).

**Conclusions:**

Based on the results, it is concluded that nutraceutical supplementation improved semen parameters in Quarter Horse stallions.

## Graphical abstract

This study aimed to evaluate the effect of nutraceutical supplementation on semen quality in Quarter Horse stallions. Sperm concentration (10x^6^) was significantly higher with supplementation (339.4 ± 17.5 sperm/mL) than without supplementation (224.6 ± 19.9). Sperm abnormalities (%) were significantly (*p* < 0.05) lower with supplementation (14.3 ± 0.6) than without supplementation (19.1 ± 0.4). Sperm kinematic parameters were significantly better with supplementation (*p* < 0.05). Based on the results, it is concluded that nutraceutical supplementation improved semen parameters in Quarter Horse stallions.

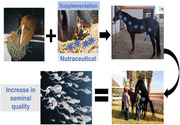

## 1 INTRODUCTION

Artificial insemination (AI) is one of the most widely used biotechnologies in equine reproduction (Aurich, 2012). AI allows for eliminating geographic barriers and allowing sport stallions within breeding programmes to breed and compete simultaneously (Alvarenga et al., 2016; Wilson & Flesner, 2017). The use of fresh, chilled, or refrigerated and frozen‐thawed semen offers many advantages to breeders as a genetic enhancer; however, in practice, many of the stallions produce sperm that is unable to withstand these processes (Grady et al., 2009; Houssou et al., 2020; Monteiro et al., 2019). Brinsko et al. (2000) suggested the term ‘bad coolers’ or ‘bad freezers’ as appropriate for stallions that exhibit a sudden decline in motility or fertility. It has been shown that sperm can produce reactive oxygen species (ROS) that are involved in various physiological events, such as sperm capacitation, if they are controlled (Aitken & Baker, 2004). However, when there is an imbalance in ROS due to oxidative stress (Del Prete et al., 2018; Halliwell & Whiteman, 2004), it produces alterations that modify semen homeostasis (El Sisy et al., 2016), affecting fertility (Freitas & De Oliveira, 2018; Lançoni et al., 2018).

Several recent studies have mentioned that semen quality efficiency is improved by reducing oxidative stress damage (Aurich et al., 2020; Aurich, 2008). Using of antioxidants directly in semen or antioxidant supplementation of stallions has improved sperm quality (Brinsko et al., 2005; Campos et al., 2020; Deichsel et al., 2008; Freitas et al., 2016). Antioxidants delay, prevent or eliminate oxidative damage to a target cell (Govindaraj et al., 2017). Several substances can help optimize some metabolic pathways (Arruda et al., 2010). In this sense, it has been observed that supplementation based on nutraceuticals containing antioxidants improves semen quality (Freitas et al., 2016). Several studies have focused on investigating the effects of different dietary compounds on semen quality. However, each one has analysed each antioxidant or nutraceutical separately. Therefore, this study hypothesized that supplementation with various antioxidants of nutraceutical origin would improve semen quality in Quarter Horse stallions. The purpose of this study was to evaluate the effect of nutraceutical supplementation with β‐carotene, lutein, lycopene, casein, selenium, docosahexaenoic acid (DHA, omega‐3), l‐carnitine, vitamin A, vitamin C and vitamin E on semen quality in Quarter Horse stallions.

## 2 MATERIALS AND METHODS

### 2.1 Location and management of stallions

The experiment was conducted during May–August 2022 in the Comarca Lagunera, in northeastern Mexico (latitude 25°23′ N and longitude 104°47′ W), under natural photoperiod conditions (spring and summer). The average annual precipitation is 230 mm, and the average temperature is 24°C, with a maximum of 41°C and a minimum of −1°C in summer and winter, respectively. The day has 13 h and 44 min at the summer solstice and 10 h and 33 min at the winter solstice (INIFAP, 2017). Six Quarter Horse stallions, aged 9–12 years and with a body condition of 5 (scale 1–9) according to the classification proposed by Carroll and Huntington (1988), were used for the experiment. These animals were housed in 4 × 4 m stalls with floor and sawdust bedding with controlled access and a 6 × 4 m sunny area with a dirt floor. The stallions were exercised thrice weekly with forced exercise and electric treadmills for approximately 30 min. The diet of the six stallions was based on alfalfa with an intake of 20% MS and 1 kg concentrate containing 14% crude protein, which was mixed with 30 g of nutraceutical. Free access to water was provided for 2 months.

### 2.2 Experimental design

Six clinically healthy stallions with proven fertility and an age range of 9–12 years were used. The stallions were evaluated before and after supplementation with nutraceutical. To assess the actual quality of the semen, semen was collected three times a day, every 2 days, on two occasions. Semen was collected every 20 days for 2 months before supplementation. Subsequently, the six stallions were supplemented with Stallion Force nutraceutical mixed with the concentrate at 30 g/day of the nutraceutical containing β‐carotene, lutein, lycopene, casein, selenium, DHA (omega‐3), l‐carnitine, vitamin A, vitamin C, vitamin E for 15 days as an adaptation period. From day 16, semen was collected every 20 days for 2 months. An artificial vagina (VA), the Botucatu model, was used for semen collection.

Immediately after collection, it was placed in a water bath at 37°C; the gel fraction was removed by filtration using an equine semen filter (Animal Reproduction Systems). On the basis of sperm concentration and semen kinematic parameters, total motility (MT%), progressive motility (MP%), rectilinear velocity (VSL μm/s), trajectory velocity (VAP μm/s) and curvilinear velocity (VCL μm/s). It was evaluated by extracting a sample of approximately 2.7 μL of semen and placing it on a slide, after which semen motility was analysed with respect to five digital images of various fields at a temperature of 37°C in a computer‐aided semen analyser (HC‐B028V). Parameters were evaluated using custom configurations for equine sperm.

The morphology was evaluated with eosin–nigrosin staining technique (RAL Diagnostics) and was used to examine sperm morphology, a 15‐μL eosin–nigrosin was mixed with an equal amount of semen on a warm and clean, and the mixture was spread gently and randomly assessed at 1000× magnification under oil immersion.

### 2.3 Statistical analysis

Experimental endpoints were analysed using an ANOVA to compare the values of the stallions fed their control vs. the same stallions fed the nutraceutical‐enriched diet. A Student's *t* test analysis was used with significance levels of *p* ≤ 0.05. All variables were analysed using the SAS (OnDemand for Academics Dashboard) programme.

## 3 RESULTS

**FIGURE 1:**
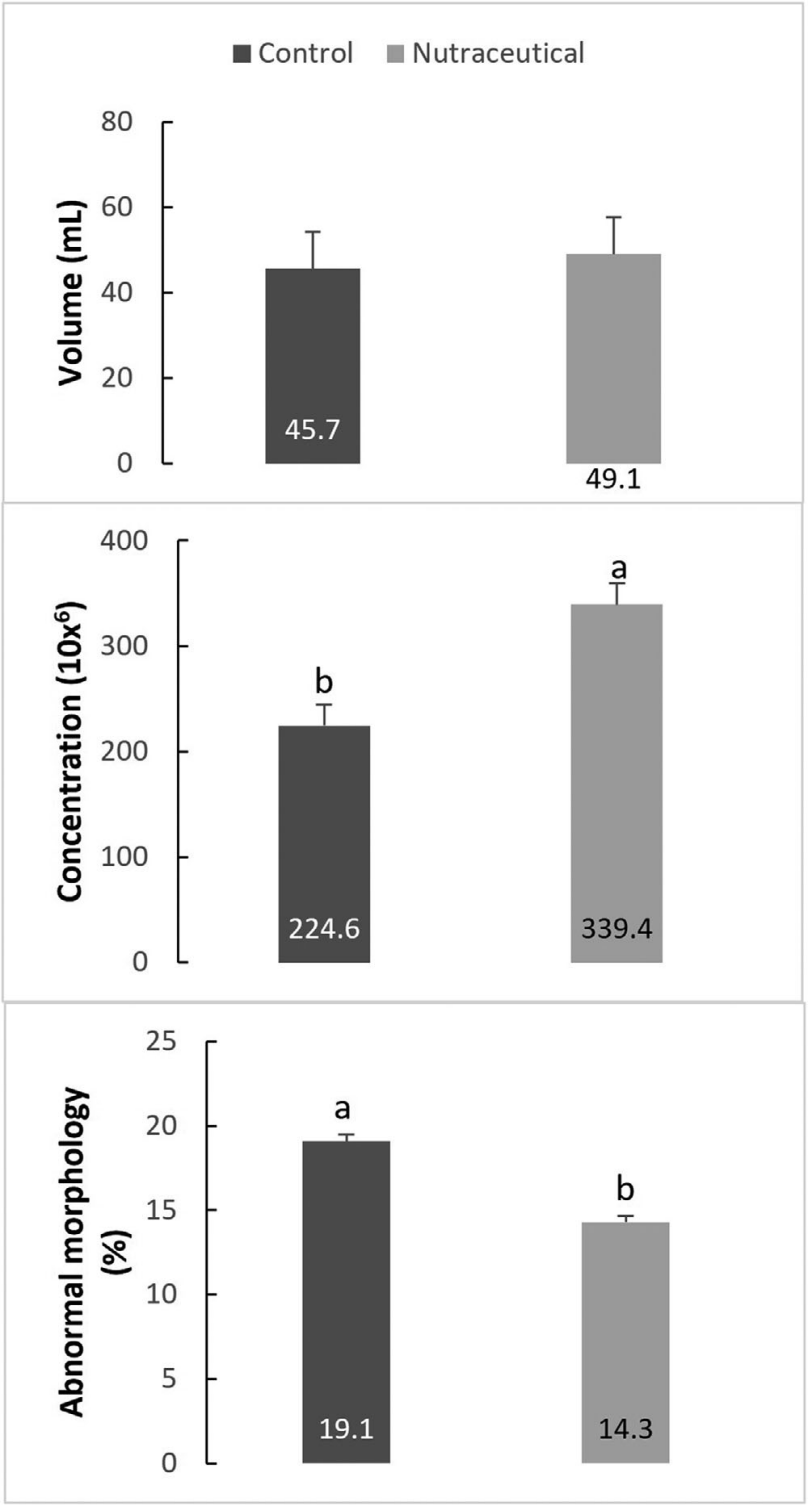
Mean ± standard error of the mean (SEM) of volume, concentration and abnormal morphology evaluated in Quarter Horse stallions without and with nutraceutical. Different superscripts (a and b) indicate a statistically significant difference (*p* ≤ 0.05).

The total average values obtained gel‐free volume during the study reported that there is no significant difference (*p* > 0.05) in the stallions before supplementation (45.7 ± 8.6 mL) and after supplementation (49.1 ± 7.8 mL). Sperm concentration was lower (*p* < 0.05) in stallions before supplementation (224.6 ± 19.9x^6^) than after supplementation (339.4 ± 17.5x^6^). Regarding sperm morphology, a significant difference (*p* < 0.05) was observed where the total number of sperm abnormalities in stallions before supplementation (19.1% ± 0.4%) decreased after supplementation (14.3% ± 0.6%) (Figure 1).

**FIGURE 2:**
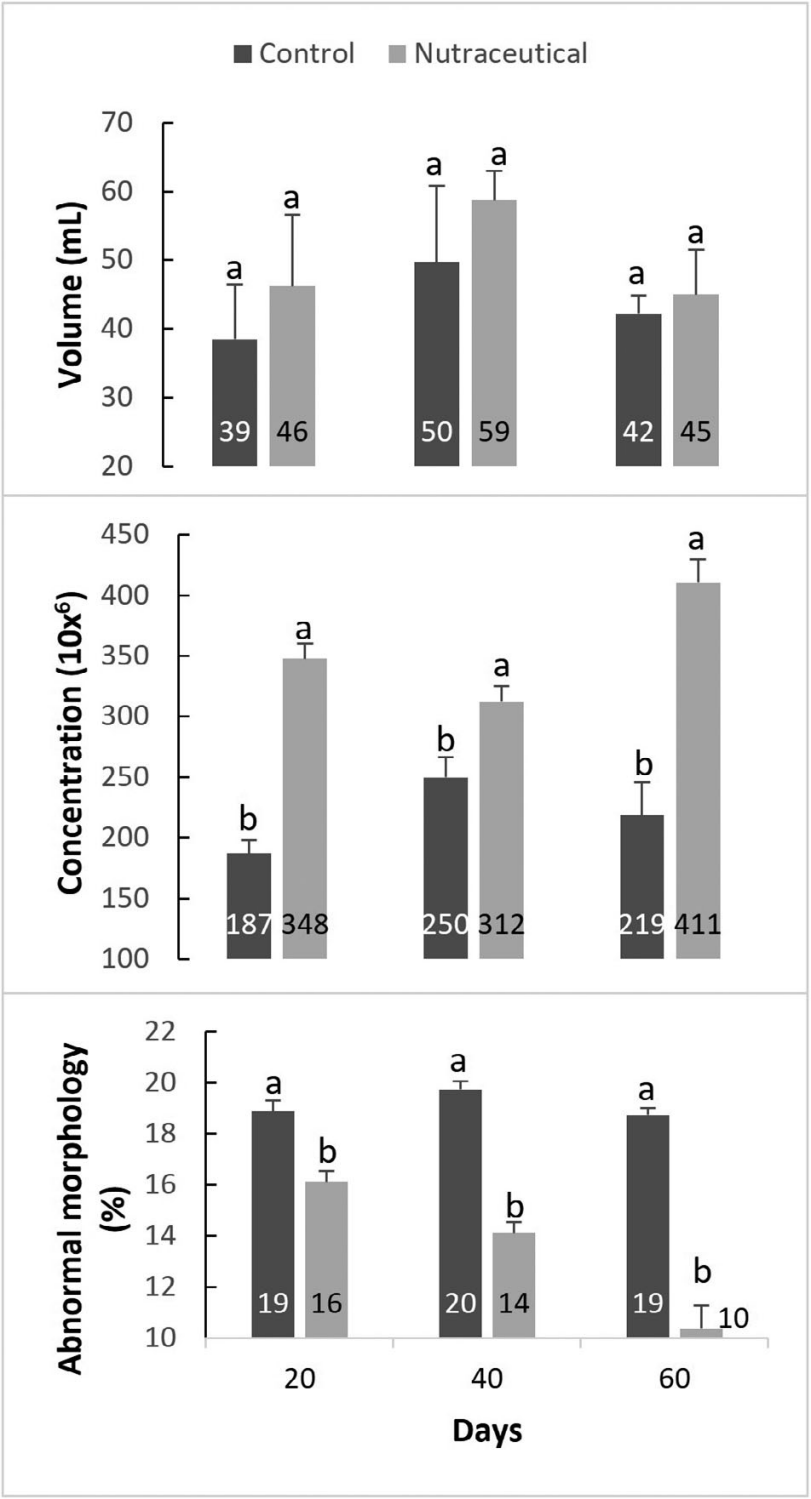
Mean ± standard error of the mean (SEM) of volume, concentration and abnormal morphology evaluated every 20 days in Quarter Horse stallions without and with nutraceutical. Different superscripts (a and b) indicate a statistically significant difference (*p* ≤ 0.05).

A statistical difference was observed (*p* < 0.05) in sperm concentration during supplementation on days 20 (187.0 ± 11.4x^6^–347.5 ± 12.5x^6^), 40 (249.8 ± 17.0x^6^–312.3 ± 13.2x^6^) and 60 (218.8 ± 27.0x^6^–410.5 ± 19.3x^6^). Regarding sperm morphology, a significant difference (*p* < 0.05) was observed over time, day 20 (18.9 ± 0.4%–16.1 ± 0.4%), day 40 (19.8 ± 0.3%–14.1 ± 0.4%) and day 60 (18.8 ± 0.3%–10.4 ± 0.9%) (Figure 2).

The kinematic parameters, total motile spermatozoa (MT%), progressive motility (MP%), rectilinear velocity (VSL μm/s), trajectory velocity (VAP μm/s) and curvilinear velocity (VCL μm/s) of the stallions improved (*p* < 0.05), with supplementation were compared to the absence of supplementation, respectively (Table 1).

**TABLE 1:** Means ± standard deviation (SD) of kinematic parameters of fresh semen kinematic parameters of ejaculates collected from Quarter Horse stallions (*n* = 6) without nutraceutical and with nutraceutical supplementation.

Variable	Control	With nutraceutical
MT (%)	79.2 ± 4.8^b^	92.3 ± 2.5^a^
MP (%)	38.1 ± 6.5^b^	68.8 ± 5.3^a^
VSL (μm/s)	67.4 ± 4.8^b^	92.8 ± 2.1^a^
VAP (μm/s)	58.5 ± 4.0^b^	90.1 ± 2.1^a^
VCL (μm/s)	116.6 ± 10.8^b^	180.6 ± 6.7^a^

Abbreviations: total motile spermatozoa (MT %), progressive motility (MP %), rectilinear velocity (VSL μm/s), trajectory velocity (VAP μm/s) and curvilinear velocity (VCL μm/s). Different superscripts (a, b) indicate a statistically significant difference (*P* ≤ 0.05).

## 4 DISCUSSION

The intakes of nutrients, mainly antioxidants, are fundamental for reproductive functions (Bazzano et al., 2021). The results of this study show the importance of nutraceutical in the production and function of spermatozoa in stallions. It can be observed that after supplementation with nutraceutical, the sperm concentration increases compared to before supplementation (339.4 ± 17.5x^6^ vs. 224.6 ± 19.9x^6^ *, p* < 0.05), which agrees with what was reported by Contri et al. (2011), who supplemented with antioxidants. A drop in testicular oxygen supply can affect the fertility of males. Some researchers have suggested that long periods of intratesticular hypoxia could be part of the aetiology of certain spermatogenic failures (Sharma et al., 2021). Apoptosis of primary spermatocytes and spermatogonia, induced by hypoxia, may contribute to the loss in this population of cells due to the large distance of oxygen diffusion from the blood vessels to the luminal region of the seminiferous tubules (Govindaraj et al., 2017). This may be mainly because some antioxidants in high concentrations in the epididymis provide energy to the sperm by helping mitochondrial β‐oxidation and increasing sperm concentration (Mongioi et al., 2016). In addition to this, lycopene, an antioxidant carotenoid concentrated mainly in the prostate and testicles, has functions in growth, regulation and protection against cellular lipid peroxidation (Imran et al., 2020).

When sperm morphology was evaluated, it was observed that abnormalities decreased after supplementation concerning the data obtained before being supplemented (14.3 ± 0.6% vs. 19.1 ± 0.4%, *p* < 0.05). These results agree with studies on Shetland ponies (Deichsel et al., 2008) and Andalusian stallions (Ruiz et al., 2021). Other studies have observed an increase in the percentage of abnormal sperm in conditions of hypoxia, where the predominant anomalies are head malformations, unlike tail anomalies that are more frequent in normal conditions (Dehdari et al., 2023). Exogenous antioxidants function as electron donors, avoiding an oxide‐reduction chain sacrificing their own molecular integrity, through three levels of protection: Prevention: consists of the formation of ROS above normal levels of the organism. Interception: interrupting the chain reaction, trapping the ROS and reducing them by forming non‐radical end products. Repair: removing biomolecules that have been damaged by free radicals (Saraswat et al., 2014). In this regard, essential micronutrients such as vitamin E, selenium (Se), vitamin A, vitamin C, zinc, copper, iron, manganese and casein are involved in the body's antioxidant defences, protecting cells from oxidative stress, mainly lipid peroxidation that alters the structure and function of the sperm membrane (Campos et al., 2020). Kinematic parameters reflect the ability of sperm to migrate through the mare's reproductive system for fertilization (Alvarenga et al., 2016). In this study, an increase (*p* < 0.05) was observed after supplementation with nutraceutical, which agrees with what was reported in Colombian stallions (Usuga et al., 2017), Quarter Horses (Nery et al., 2020), Dare‐Shuri (Bahrami et al., 2020) and Arabs (El Sisy et al., 2016), this can be attributed to the fact that antioxidants such as l‐carnitine provide energy to sperm by increasing the transport of fatty acids to the mitochondrial matrix (Mongioi et al., 2016). However, in other studies, the kinematic parameters did not increase after nutraceutical supplementation (Alamaary et al., 2019; Brinsko et al., 2005; Ravi et al., 2016). In a study carried out on Mangalarga Marchador stallions, they reported that there was no statistical difference in rectilinear speed (Rodrigues et al., 2017).

## 5 CONCLUSIONS

In conclusion, semen parameters such as sperm concentration, morphology and motility were significantly improved in the stallions after supplementation with the nutraceutical. However, it is necessary to perform further experiments involving a larger number of animals and evaluate the semen during the cooling and freezing process of the spermatozoa.

## AUTHOR CONTRIBUTIONS

*Development of methodology; preparation of the draft (first version); collection; analysis and interpretation of data*: Edgar Díaz‐Rojas. *Formal analysis; supervision; validation; visualization of the writing of the manuscript*: Dalia I. Carrillo‐Moreno. C*onceptualization; formal analysis; visualization; review and editing*: Viridiana Contreras‐Villarreal. *Funding acquisition; supervision; visualization*: Fernando Arellano‐Rodríguez*. Conceptualization; data curation; supervision; validation: Alan S. Alvarado‐Espino. Validation; visualization; writing—review and editing; approval of the final version to be published*: Oscar Ángel‐García.

## CONFLICT OF INTEREST STATEMENT

All authors declare that there is no conflict of interest that could affect the integrity of the currently reported results.

## FUNDING INFORMATION

None.

## ETHICS STATEMENT

All methods and handling of the stallions were in strict accordance with international (FASS, 2010) and national (NAM, 2002) guidelines for the ethical use, care and welfare of animals in research, with institutional approval number UAAAN‐UL/ 037/22‐CA‐MV‐LN.

### PEER REVIEW

The peer review history for this article is available at https://publons.com/publon/10.1002/vms3.1289.

## Data Availability

The data that support the findings of this study are available from the corresponding author upon reasonable request.
